# Indiana State Medical Society

**Published:** 1858-06

**Authors:** Thos. W. Fry

**Affiliations:** Crawfordsville, Ind.


### SKETCH OF THE PROCEEDINGS OF THE INDIANA STATE MEDICAL SOCIETY.

FURNISHED BY THOS. W. FRY, M.D., CRAWFORDSVILLE, IND.

The society convened on the 19th of May, in Indianapolis, in the hall of the House of Representatives.

Dr. Bullard, the president, being in Europe, Dr. Cogley, one of the vice presidents, took the chair; Drs. Elliott and Newcomer, secretaries. A committee of five on admissions, was appointed, and requested to report at the afternoon session.

Verbal reports were made by the secretary, treasurer, and librarian. Dr. Kitchen, from the committee on publication, reported verbally.

Adjourned till 2 o'clock p. m.

AFTERNOON SESSION.

Society met pursuant to adjournment.

The committee on admissions reported the names of twenty-two physicians for membership, all of whom were elected.

The report of the committee on ethics was made special order of the day for 3 o'clock p. m.

Dr. Humphreys, from the committee on the practice of medicine, stated that a report would be presented at some future time.

In the absence of the chairman of the committee on surgery, Dr. Lomax was requested to read some cases which he had written out.

Dr. Ayres, chairman of the committee on obstetrics, made a verbal report, and on motion the committee was continued, to report at the next annual session.

The committee on chemistry reported verbally, through their chairman, Dr. Parvin, and on motion was discharged.

Committee on medical education called; the chairman absent; on motion, Drs. Fishback and Brower were added to the committee, and their report made the special order of the day for to-morrow, 10 o'clock a. m.

No report from the committee on materia medica.

Dr. Hutchinson, from the committee on microscopy, read a very excellent report, which was received and referred to the committee on publication.

Committee on chronic diseases of the brain called, and their report made the special order of the day for to-morrow morning, 11 o'clock.

Dr. Meeker continued his report from last session on fractures and false joints—a superior paper. Received and referred to the committee on publication; committee continued.

Dr. Knepfler read a good paper on the history, the use and abuse of mercury. Referred to the publication committee.

Adjourned till half-past 7 p. m.

WEDNESDAY MORNING.—Dr. Lomax read two remarkable and very interesting cases as a part of the report on surgery. One of them will attract the attention of medical philosophers in all parts of the world. It was a case in which there was a fracture of the skull, and a large portion of brain lost, producing very strange and wonderful moral and mental developments. It will appear in our transactions.

Dr. Newcomer, from the committee on substitutes for quinine in the treatment of intermittents, reported verbally, that no substitute for severe cases had as yet been discovered.

The chair announced the names of Drs. Brower, Parvin, Humphreys, Meeker, and Woodworth, as a committee on nominations.

Report of the committee on ethics indefinitely postponed, on motion of Dr. Brower.

The special order of the day for 10 o'clock, the subject of medical education, was then taken up. Dr. Fishback read an admirable report. There was general discussion of the subject by Drs. Brower, Fishback, Fry, Lomax, Ayres, Harding, Cornett, and Jameson, and a deep interest was manifested by the society.

The hour for the report of the committee on chronic diseases of the brain having been announced, the discussion was postponed. Dr. Elliott, chairman of the committee, read his report, which was received and referred.

The chair announced the following standing committees:

Executive Committee—Drs. Dunlap, Woodburn, Bullard, Thompson, and Kitchen.

Committee on Finance—Drs. Mauzy, R. C. Moore, Bullard, Knepfler, and Darrach.

Committee on Publication—Drs. Parvin, Darrach, Elliott, Bullard, and Jameson.

Committee on Ethics—Drs. Woodworth, Lomax, Humphreys, and Murphy.

Committee on Chronic Diseases of the Brain—Drs. New, Lomax, and Harding.

Committee on Practice of Medicine—Drs. Woodworth, Ayres, and Sutton.

Committee on Surgery—Drs. R. Spencer, Latta, and Lomax.

Committee on Obstetrics—Drs. Ayres, Harding, and J. Ellis.

Committee on Medical Education—Drs. W. R. Smith, Newland, Fry, and Fishback.

Committee on Materia Medica—Drs. Jameson, Fishback, and Brower.

AFTERNOON SESSION.—Committee on fee bill reported; report received and laid upon the table.

On motion of Dr. Fishback, Dr. Fry was appointed to report on medical inhalation at the next session.

Dr. Brower, chairman of the committee on nominations, reported as follows:

*President*—Dr. Nathan Johnson.
*Vice Presidents*—Drs. Austin, Fry, Newland, and Latta.
*Corresponding Secretary*—Dr. J. M. Gaston.
*Recording Secretaries*—Drs. T. B. Elliott and F. S. Newcomer.
*Treasurer*—Dr. Charles Parry.
*Librarian*—Dr. Wm. P. Thompson.

Delegates to the American Medical Association:

Drs. Ayres, Woodburn, Kitchen, Jameson, W Davidson, S. Davis, W. T. S. Cornett, Sloan, Austin, Town, Bowman, Clapp, jr., Parvin, J. J. Wright, Mauzy, Hillis, Boynton, Thompson, Tinton, Knepfler, Humphreys, Latta, R. Spencer, Casselberry, New, Jessup, Fishback, DeBruler, Maxwell, Grimes, Ford, Ray, Graff, Harding, Brower, Freeman, Sutton, Elliott, Bobbs, T. B. Harvey, Hutchinson, Dunlap, Coleman, Fry, Haughton, Woodworth, and O'Farrall.

Dr. Woodburn read the report of a case of dry gangrene.

Dr. Humphreys reported a plan for an interchange of public transactions of local societies within the U. S.

Discussion on medical education resumed, and after a large discussion, the report was laid on the table on motion of Dr. Hillis.

Dr. Newcomer offered the following resolution:

*Resolved*, That a committee of three be appointed to report at its next meeting, on the history of uses and abuses of Tobacco; adopted. Committee—Drs. Wm. Spencer, Brower, and Smith.

Dr. Brower offered the following resolution:

*Resolved*, That this society receive, with cordial approbation, the circular emanating from Rush Medical College at Chicago, upon the subject of a reform in the system of public medical instruction, and pledge themselves, so far as may be in their power and within the sphere of their proper function, to extend their cordial co-operation in all measures touching the purification and improvement of our system of education.

On motion of Dr. Haughton, a committee was appointed to report on the treatment of syphilitic diseases without the use of mercury; carried.

A vote of thanks, tendered the state librarian for the use of the Hall.

On motion, adjourned to meet the third Tuesday in May, 1859.

T. G. COGLEY, *President*.

T. B. ELLIOTT,

